# Consequences of COVID-19 on the Reindeer Husbandry in Norway: a Pilot Study Among Management Staff and Herders

**DOI:** 10.1007/s10745-021-00295-0

**Published:** 2022-05-02

**Authors:** Guro Lovise Hole Fisktjønmo, Marius Warg Næss

**Affiliations:** Norwegian Institute for Cultural Heritage Research (NIKU), Tromsø, Norway

**Keywords:** Reindeer herding, Pandemic, COVID-19, Mobility, Pastoral management, Norway

## Abstract

The outbreak of COVID-19 has had an enormous impact on most of society. The most effective measure to prevent the spread has been reducing mobility, which is especially problematic for pastoralists relying on mobility to follow the movement of their livestock. We investigated to what degree Norwegian reindeer husbandry and the reindeer husbandry management system are affected by COVID-19 and government restrictions to mitigate the effects of the pandemic. For reindeer herders, our main finding was that the COVID-19 had little to no impact on their daily work. However, impacts varied by domain, with work in corrals, income, and slaughter being negatively affected. For employees in the management system, communication/contact with herders and visits/control of corrals/slaughter have been negatively affected. Employees in the management system were satisfied with how information concerning COVID-19 and prevention measures have been communicated by the central government, while the herders were mainly dissatisfied.

**Supplementary Information:**

The online version contains supplementary material available at 10.1007/s10745-021-00295-0.

## Introduction

### COVID-19 and Pastoralism

The highly infectious coronavirus disease (COVID-19) has been declared a global pandemic by the World Health Organization (WHO). As of July 2021, the virus has claimed over 4 million^1^ lives worldwide and continues to spread around the globe.

Restricting mobility and social distancing have proven to be effective measure to control the spread of COVID-19 (Kissler et al., 2020). Nomadic pastoralists, who are highly mobile for daily herding of their livestock, are thus at higher risk of a rapid spread of COVID-19 (Egeru et al., 2020; Griffith et al., 2020; Yousuf et al., 2020) so that the management of the pandemic amongst pastoralists is especially challenging (Griffith et al., 2020). For example, among pastoralists in the Greater Horn of Africa, Griffith et al. (2020) found that COVID-19 control efforts negatively impact livelihoods and food, income, and nutrition security. Consequently, they recommend targeted public health measures to protect market access and ensure mobility for pastoralists. This is supported by nomadic pastoralists in Mauritania, who state that they face an unprecedented situation due to closed national borders and movement restrictions (Food and Agricultural Organization (FAO) of the United Nations 2020). Mohamed et al. (2020) found that for pastoralists in five different countries in Asia, Africa, and Europe, infection control measures lead to restricted mobility and market engagement, resulting in an intensified pattern of social differentiation. Among pastoralists in Somaliland, the pandemic has imposed additional economic losses to livelihoods already weakened by import bans on livestock (Mtimet et al., 2021). Another reason why pastoralist communities are significantly vulnerable to the pandemic is the role of elders who hold much traditional knowledge among pastoralists in the Greater Horn of Africa but at the same time are the age group most likely to succumb to a disease-like COVID-19 (Griffith et al., 2020).

## The Status of COVID-19 in the Reindeer Husbandry in Norway

The first cases of COVID-19 in Norway were detected in mid-February 2020, leading public health authorities to provide advice on infection control measures, such as hand hygiene, sneezing and coughing habits, isolation of individuals with symptoms, and tracing of contacts of confirmed cases. Further, people were advised to avoid unnecessary travel and to work from home if possible. On the 12^th^ of March, the government issued stricter measures and instituted quarantine for those who entered the country. The same day, the Government closed all kindergartens and schools, in addition to several health and wellness companies, and banned all cultural events and all organized sports (Helsingen et al*.,* 2020). Moreover, restaurants, coffee shops, and pubs were closed if 1 m distance could not be maintained between the customers (Ursin et al*.,* 2020, table 1).

**Table 1:** Distribution of sample size and response rate in this study. *Sample size* refers to the number of people responding to the survey, while *response rate* refers to the number of people responding to questions pertaining to this particular topic

	**Sample size**	**Response rate**
**Herders**		
Affect husbandry		
Overall	17	16
Elaborated	17	11
Different areas	17	16*
Information from management	17	16
Elaborated	17	7
Financial support	17	14
Elaborated	17	8
Infection control measures	17	14
Elaborated	17	13
**Management**		
Affect husbandry	9	9
Affect management	9	9
Information from management	9	8
Elaborated	9	2
Infection control measures	9	7

^*^One participant only answered the question related to income

The following year, Norwegian society experienced several openings and lockdowns depending on fluctuations in viral load. During this time, active discussions were conducted in the national press and other media concerning the costs and benefits of preventive measures. However, little information has been provided concerning the effect on pastoral communities in Norway, highly mobile reindeer herders who, by following the seasonal movements of their herds (Paine, 1994:14), expose themselves to different challenges than other industries affected by COVID-19 related restrictions. By being classified as a primary industry, reindeer husbandry was exempted from some of the government introduced measures, including access to herding cabins and crossing national borders to follow the herds' movement (Ministry of Agriculture & Food, 2020b).

Compounding the issue is the fact that when the pandemic first hit Norway, reindeer husbandry in northern parts of Norway was already experiencing a crisis that occurred during the winter and spring of 2020 when unusually large amounts of snow severely impacted forage availability. This affected seventy-five percent of domesticated reindeer in Norway, leaving them already vulnerable (Ministry of Agriculture & Food, 2020a). Thus, the president of the Saami Parliament argued that the reindeer husbandry experienced a “double crisis year” in 2020 as a consequence of the substantial amount of snow and the COVID-19 pandemic (Keskitalo, 2020).

While the forage crisis is well documented by the Government and the County Governors (Norwegian: Statsforvalteren*)*, the effects of the COVID-19 situation and subsequent public health measures for the reindeer husbandry have had less coverage. This calls for more information about preventive measures and the consequences of the COVID-19 pandemic for reindeer herders. Elders among Saami reindeer herders have a role similar to that of pastoralist elders in the Greater Horn of Africa due to their long experience (Næss et al*.,* 2021). Furthermore, while herders may work alone for days, they are more often working with partners who are likely to change along with the seasonal regrouping of the herd (Paine, 1994:103). Herders from different groups must occasionally work together to separate herds that are mixed or retrieve animals that have wandered off (Paine, 1994:103). Thus, social distancing and infection control measures might impact day-to-day work conditions for reindeer herders.

We launched a pilot study to investigate how the COVID-19 pandemic and control measures have impacted reindeer herders in Norway and the official reindeer herding management system. For example, in the county of Nordland, all employees involved with the management of reindeer husbandry are working from home but are available by phone or email (County Governor of Nordland, 2020). However, there are no data about how this affects their work in the field, communications with the herders, and information flow between herders and the management system. This pilot study thus has a dual objective: to investigate how 1) herders and 2) employees in the management system perceive the effects of COVID-19 on the Norwegian reindeer husbandry.

## Methods

### Reindeer Husbandry

**Fig. 1:**
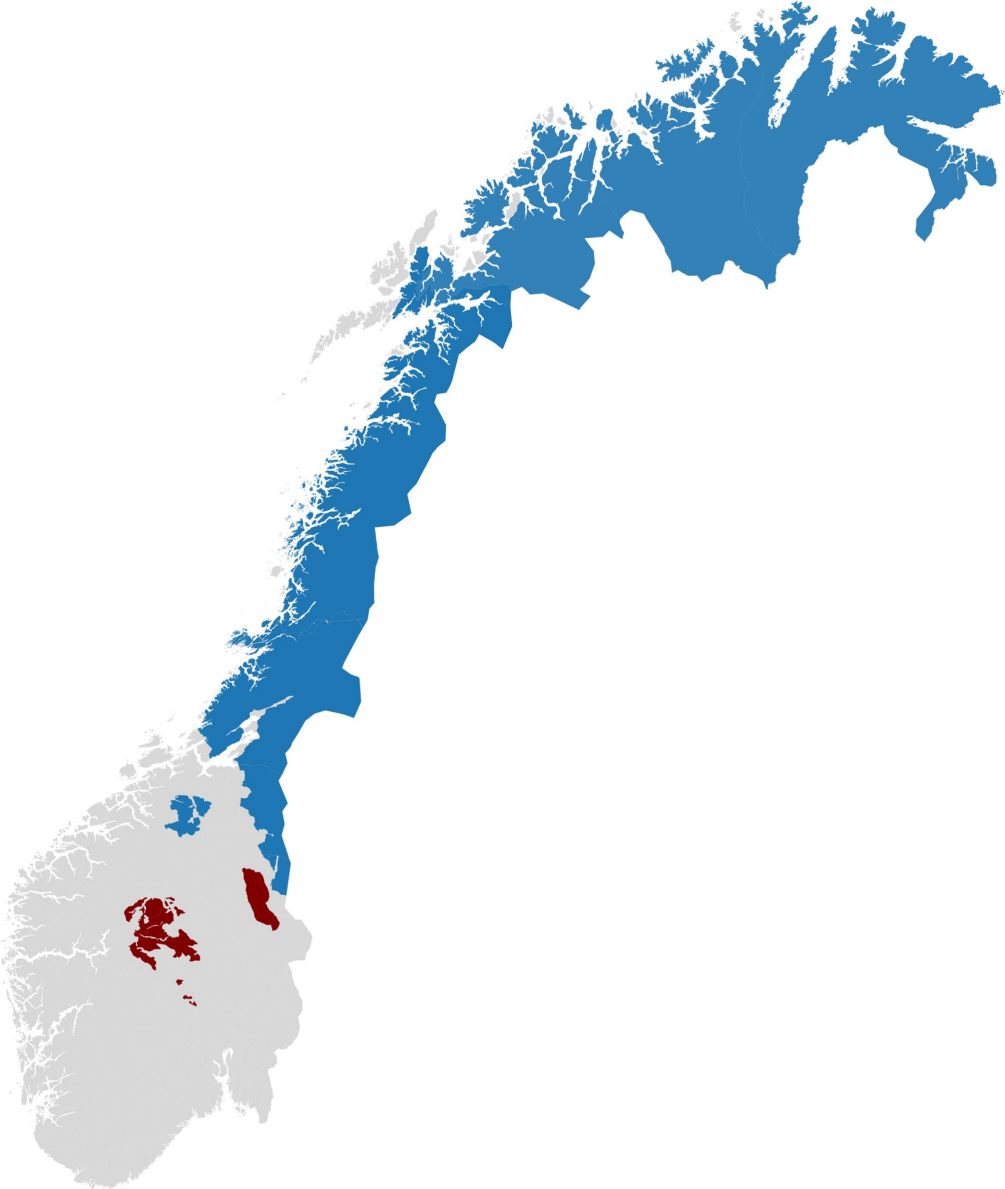
Map over reindeer herding in Norway. Saami reindeer husbandry is marked in light blue while tamreinlaga are marked in red. Map created in Python 3.6.1 (https://www.python.org/) with background map from GADM (https://gadm.org/maps/NOR.html) and official reindeer districts from NIBIO’s kilden (https://kart8.nibio.no/nedlasting/dashboard)

Reindeer husbandry in Norway exists in two different forms: 1) the Saami herders and 2) the tamreinlag (non-saami) herders. Most herders are affiliated with Saami reindeer husbandry, which is the cornerstone of indigenous Saami culture (Bostedt, 2001). It developed as a pastoral economy at least 400 years ago and probably evolved from a hunting culture based on wild reindeer (Paine, 1994; Riseth & Vatn, 2009). Saami reindeer husbandry is organized into three layers: the ‘*siida*-share’ is a license granted by the government entitling the owner to manage a herd of reindeer with a designated pasture. One or more *siida*-shares constitute a *siida*, which is a cooperative herding group of independent households traditionally organized around kinship. Finally, *siidas* are grouped into districts: formal administrative units defined by the government (cf. Næss & Bårdsen, 2013; Næss & Bårdsen, 2015; Fisktjønmo et al*.,* 2021; Næss et al*.,* 2021). From a national point of view, Saami reindeer husbandry is a relatively small industry consisting of 535 *siida*-shares and 3329 affiliated individuals (Norwegian Agriculture Agency, 2020:27). Nevertheless, Saami reindeer husbandry is vital from a local and Saami point of view in terms of economy and culture. Moreover, around 40% of Norway’s landmass is used by reindeer herders (cf. Næss & Bårdsen, 2015). The Saami reindeer pastures extend from Troms and Finnmark county in the north to Innlandet county in the south (Fig. 1).

Tamreinlag (non-Saami) herding is based on the Saami reindeer husbandry tradition.^2^ Initially, only people with a Saami heritage were permitted to practice reindeer husbandry in Norway (Norwegian: ‘reinmerke’). However, the tamreinlag are granted separate permissions in the Reindeer Husbandry Law (Norwegian: Reindriftsloven, Ministry of Agriculture and Food, 2007:§8) to herd reindeer despite lacking Saami heritage. The tamreinlag are not organized in *siida*-shares, districts, or *siidas*, but rather as higher corporate governance (e.g., a limited company). There are four tamreinlag in Norway, with pastures expanding between Innlandet, Viken, and Vestlandet (Fig. 1).^3^

### The Reindeer Husbandry Management System

According to Ulvevadet (2008:55), the management of the reindeer husbandry consists of a complex co-management system with participants from the bottom to the top. Moreover, “[…] there are three organizational systems with vertical and horizontal interaction among all its organizational parts” (ibid.). Our study targeted the administrative system that goes from the Parliament to the Ministry of Agriculture and Food and further to the Reindeer Husbandry Administration. Before 2014, the Reindeer Husbandry Administration’s main office was in Alta. It was responsible for local offices in six different reindeer husbandry areas: East-Finnmark, West-Finnmark, Troms, Nordland, Nord-Trøndelag, and South-Trøndelag/Hedmark (see Næss, 2009Appendix II for details). Local offices in each reindeer husbandry area were responsible for providing herders with assistance and advice (Ulvevadet, 2008:65).

This system was changed in 2014. While the overall administration remains under the Ministry of Agriculture and Food, the area offices, previously subsidiaries of the Reindeer Husbandry Administration in Alta, became the responsibility of the County Governors. Additionally, the Reindeer Husbandry Administration in Alta changed its name to the Directorate of Agriculture (Norwegian: Landbruksdirektoratet). The County Governor is a regional governmental administrative authority responsible for helping the government achieve its overall policy goals for reindeer husbandry in Norway, including reaching the goals of ecological, economic, and cultural sustainability (County Governor, 2020). This work includes, among other things, controlling licenses, offering advice in fencing matters, granting exemptions from the grazing rules where there are compelling grounds for doing so, and preventive measures against damage caused by predators (County Governor, 2020). Currently, the day-to-day management of reindeer husbandry is divided between Troms and Finnmark county, Nordland county, and Trøndelag county^4^ and administered through ten local offices and approximately 35^5^ employees.

### Study Design and Protocol

The research reported in this study is based on a pilot survey targeting 1) reindeer herders, and 2) employees at the Country Governors offices and Directorate of Agriculture in Alta between December 2020 and February 2021. We developed different surveys for each group: 474 copies of the herders’ survey were distributed to the district leaders of the 75 Saami districts and the four tamreinlag, and 66 copies of the management employees’ survey were distributed among 11 offices at the County Governor in Troms and Finnmark, Nordland, and Trøndelag and the Directorate of Agriculture. Each district leader/office received a letter with information concerning the survey and a request to distribute a further six enclosed surveys to other herders/employees in their district/office. Each copy of the survey contained information about the study, privacy protection, and a pre-paid envelope for returning a completed survey. The surveys to both herders and employees at the Country Governors offices and Directorate of Agriculture contained questions concerning: 1) general information about the participant, 2) consequences of the COVID-19 and the following infection control measures, and 3) open-ended questions about infection control measures, and which preventive measures participants had taken with regards to the pandemic (see S1 for the survey). The survey targeting herders focused on different aspects of reindeer related work and how they experienced the dissemination of COVID-19 related information relevant for reindeer herding. The survey targeting management system employees was more focused on how the provision of assistance and advice to herders has been impacted by COVID-19 (see S1 for the survey).

We received a total of 17 responses from reindeer herders and nine responses from employees at the offices of the Country Governors offices and Directorate of Agriculture (Table 1, see also Table 2), a relatively small response rate and a relatively large difference in the response rate between reindeer herders (3%) and employees in the management system (16%). Initially, the survey was designed as a Web survey. However, the Norwegian centre of research data (NSD) did not permit us to do this because none of the platforms available fulfilled the requirements set by NSD for online surveys.^6^ ^7^ Consequently, the survey had to be distributed by mail. While mail surveys have been reported to have higher response rates than Web surveys in general (Shih & Fan, 2008), this seems to depend on the possibility to send follow-up reminders. Follow-up reminders have shown a positive effect on postal questionnaires' response rate (Barclay et al., 2002), but several factors hindered this in our study. First, since NSD requested an anonymous survey, we had to send survey requests to district leaders among reindeer herders (as this is officially available information) and the Country Governors offices and the Directorate of Agriculture. Thus, the distribution of the survey was limited by the willingness of the official recipients to further distribute it. This is witnessed by the fact that 76% of herders responding to the survey had a commission of trust in the reindeer husbandry (e.g., as members of the district or *siida* board), and 55.5% of employees in the management system responding to the survey had a position of leadership (e.g., director and section leader). In short, most returned surveys seems to consist of individuals receiving the survey (i.e., individuals in leadership positions) and not from redistribution of the survey to other individuals. The request for anonymity also implied that we could not collect information that could be used for distributing follow-up reminders.^8^

**Table 2:** Descriptive data about the participant in the study

	**No. of participants**		**Mean age (SD)**	
	Male	Female	Male	Female
**Husbandry**	8	8	45.0 (12.8)	48.0 (14.8)
**Management**	5	4	48.6 (17.2)	49.0 (3.7)

## Results

### Consequences of COVID-19

**Fig. 2:**
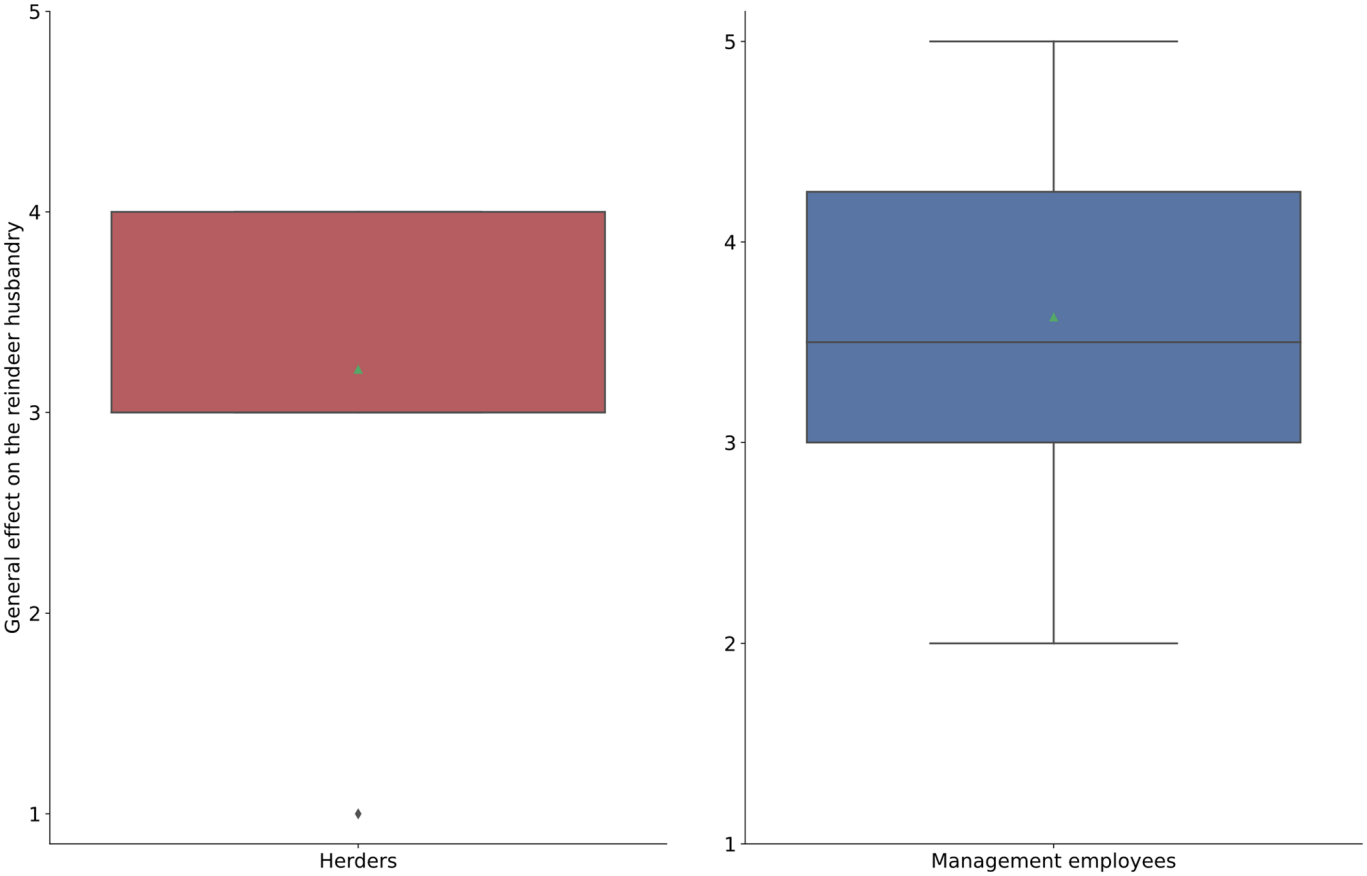
Boxplot showing how both reindeer herders and management officials rank the effect that COVID-19 have had on the reindeer husbandry. The effect is ranked on a scale from 1 to 5 where, 1 = positive effect and 5 = negative effect (Q9, S1.2 for employees in management system & Q11, S1.1 for herders)

**Fig. 3:**
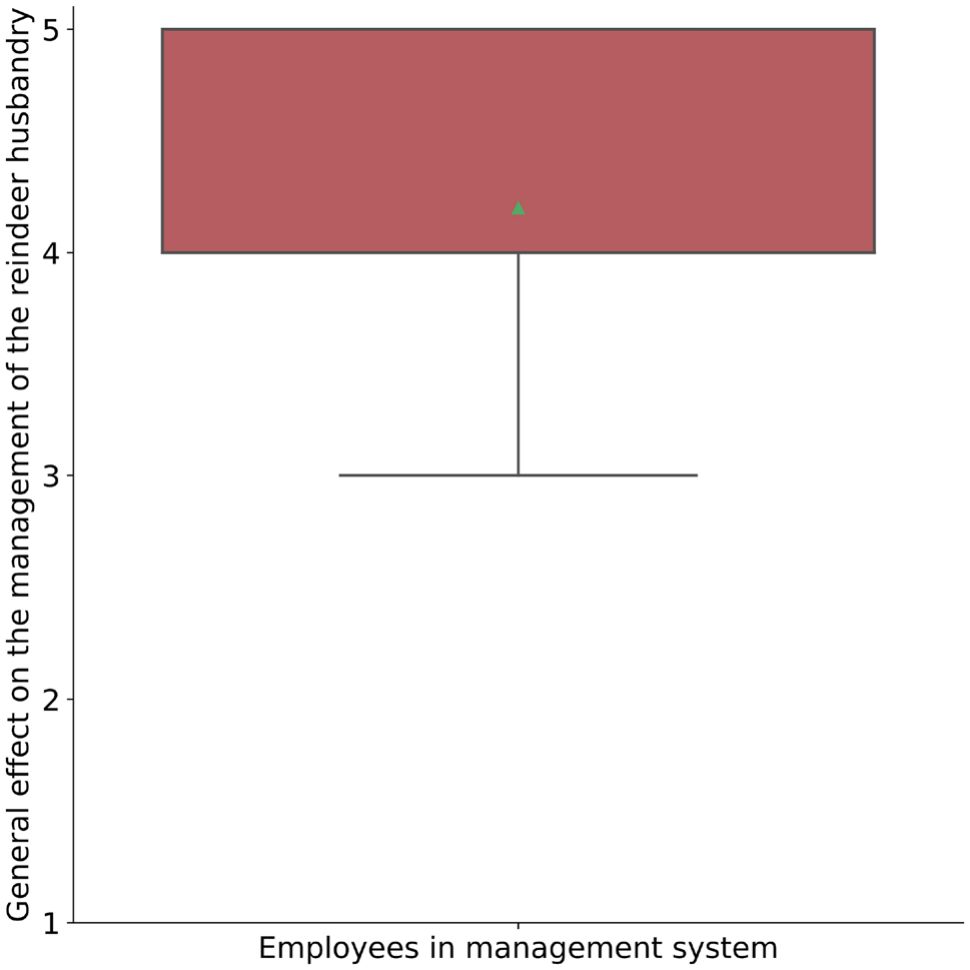
Boxplot showing how employees in the management system rank the effect that COVID-19 have had on the management of the reindeer husbandry. The effect is ranked on a scale from 1 to 5 where 1 = positive effect and 5 = negative effect (Q10, S1.2)

When asked to rank the overall consequences of COVID-19 on the reindeer husbandry on a scale from 1 to 5 (where 1 is a very positive impact and 5 is a very negative impact), herders reported that the effects were slightly negative (3.2 ± 0.8 SD, 14 herders ranked the effects from 1–5, two herders reported no effects, and one did not respond, Q11, S1.1) and employees in the management system ranked the effects as a bit more negative (3.6 ± 1.1 SD, eight employees ranked the effects from 1–5, one reported no effects, Q9, S1.2) (Fig. 2). When asked to rank the overall consequences of COVID-19 on the management system, employees reported that the effects were negative (4.2 ± 0.8, five employees ranked the effects from 1–5 while four reported no effects, Q10, S1.2) (Fig. 3).

**Fig. 4:**
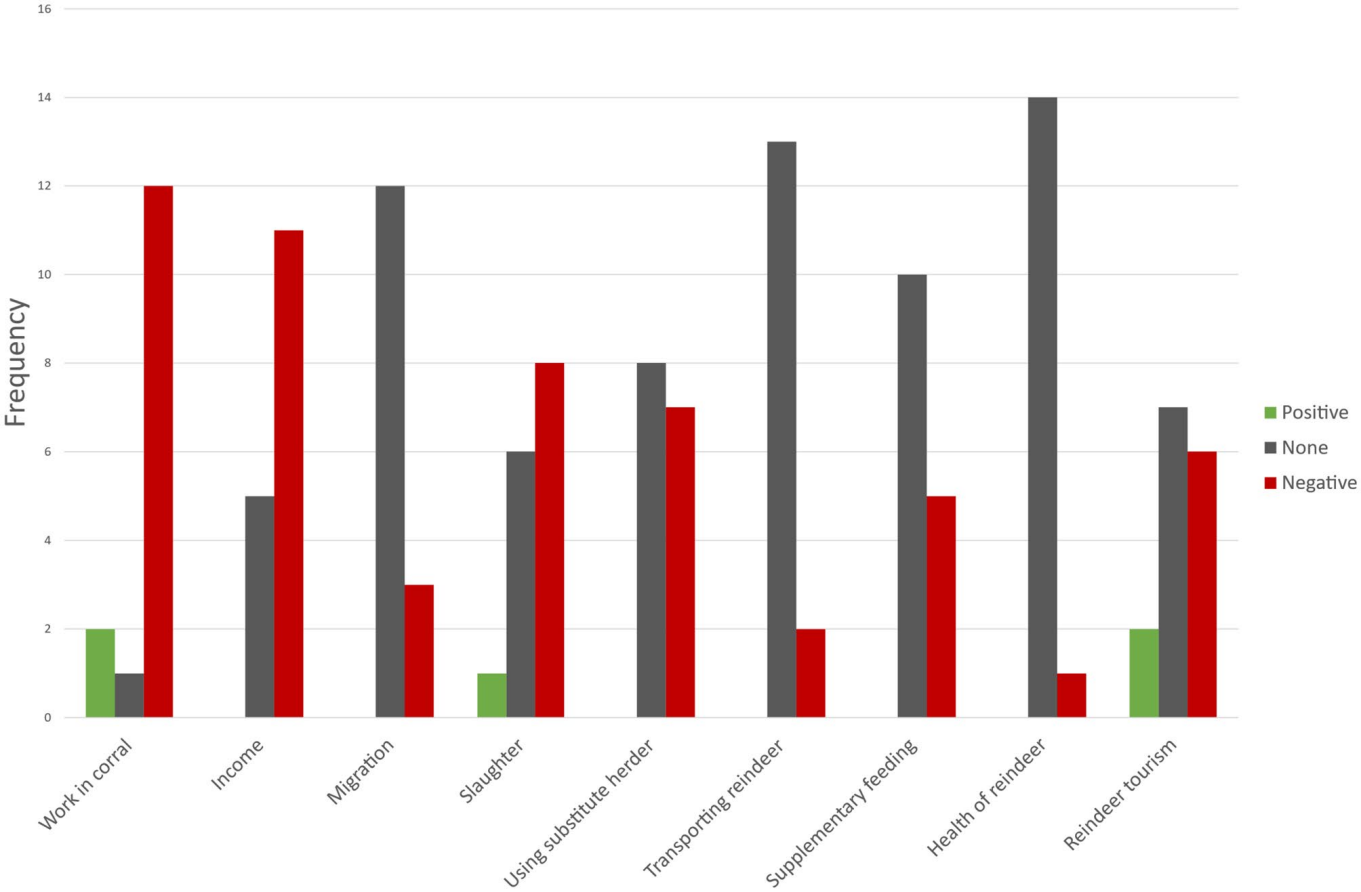
The frequency of how herders have ranked the effect COVID-19 on different areas of the reindeer husbandry. The scale 1 = very positive and 2 = some positive has been recoded to ‘positive; 4 = some negative and 5 = very negative has been recoded to negative. Based on Q13, S1.1

When herders were asked how COVID-19 affected day-to-day work in different areas of the reindeer husbandry (Q13, S1.1), almost no area was reported to be affected positively (across all areas, the sum of positive and very positive was 5), more areas were reported to be negatively affected (across all areas the sum of negative and very negative was 51). Yet, across all areas, COVID-19 had little effect on day-to-day work (across all areas, the sum was 76, Table 3). However, different areas seem to be differently affected: when recoding ‘some positive’ and ‘very positive’ to ‘positive’ and ‘some negative’ and ‘very negative’ to ‘negative’ (based on Table 3), work in corrals, income, and slaughter are ranked as being negatively affected while using a substitute herder, transporting reindeer, supplementary feeding, and the health of reindeer are ranked as being not affected at all (Fig. 4).

**Table 3:** Reindeer herders’ response concerning how COVID-19 has affected different areas of the reindeer husbandry (Q13, S1.1)

	**Very positive**	**Some positive**	**None**	**Some negative**	**Very negative**	**n**^**a**^
**Work in corral**	1	1	1	8	4	15
**Income**	0	0	5	5	6	16
**Migration**	0	0	12	2	1	15
**Slaughter**	0	1	6	6	2	15
**Using substitute herder**	0	0	8	3	4	15
**Transporting reindeer**	0	0	13	2	0	15
**Supplementary feeding**	0	0	10	5	0	15
**Health of reindeer**	0	0	14	0	1	15
**Reindeer tourism**	1	1	7	0	6	15
**Total**	2	3	76	31	24	

^a^Response rate

**Fig. 5:**
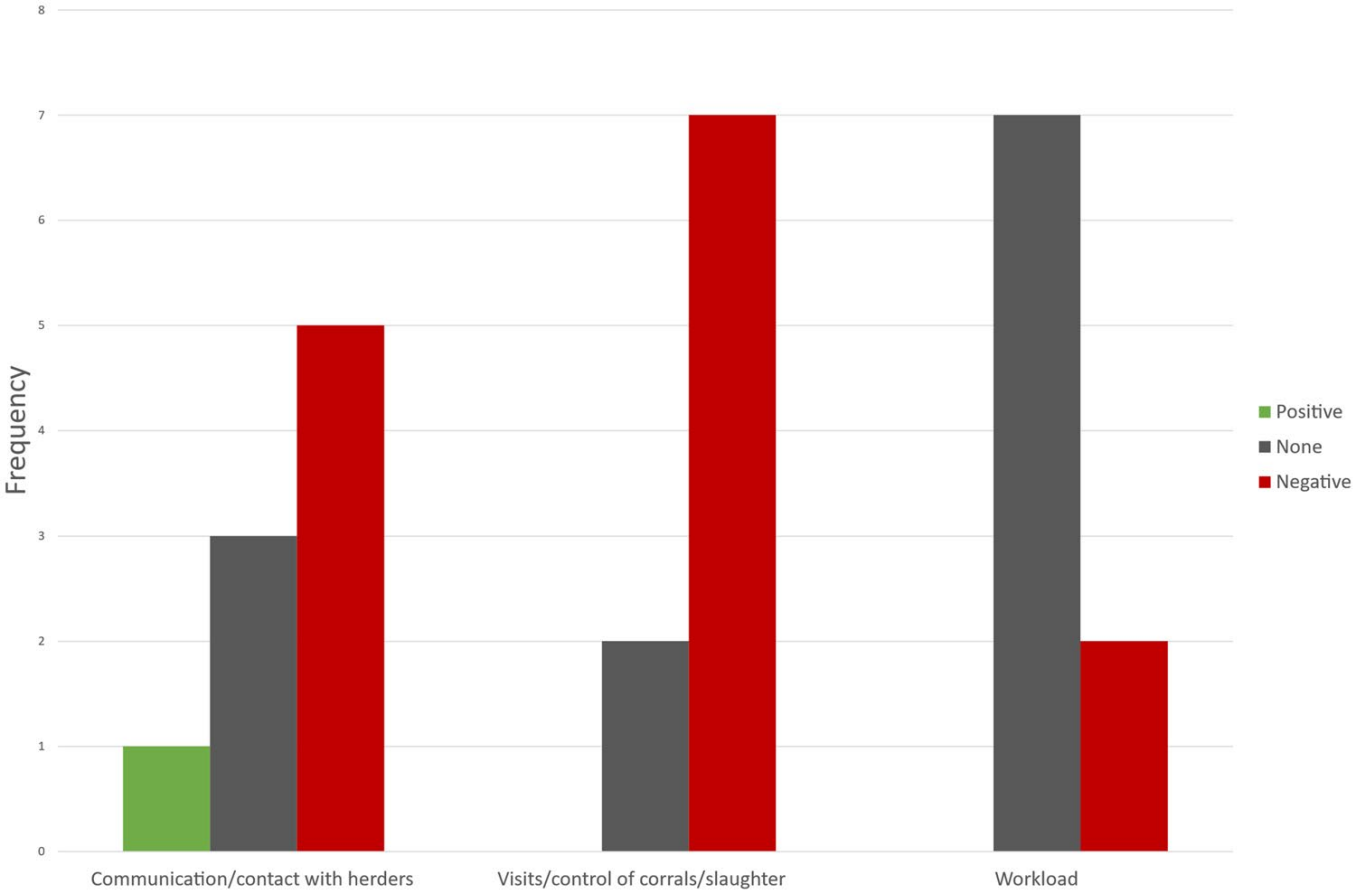
The frequency of how employees in the management system have ranked the effect COVID-19 on their day-to-day work. The scale 1 = very positive and 2 = some positive has been recoded to ‘positive; 4 = some negative and 5 = very negative has been recoded to negative. Based on Q12, S1.2

When employees in the management system were asked how COVID-19 affected day-to-day work (Q12, S1.2), almost no area was reported to be affected positively (across all areas, the sum of positive and very positive was 1). More areas were reported as not being impacted (across all areas the sum was 12), but across all areas COVID-19 had a negative effect on day-to-day work (across all areas, the sum was 14, Table 4). When recoding ‘some positive’ and ‘very positive’ to ‘positive’ and ‘some negative’ and ‘very negative’ to ‘negative’ (from Table 4), communication/contact with herders and visits/control of corrals/slaughter are ranked as being negatively affected. In contrast, the workload is ranked as being not affected at all (Fig. 5).

**Table 4:** Employees in the management response concerning how COVID-19 has affected different areas of the management system (Q12, S1.2)

	**Very positive**	**Some positive**	**None**	**Some negative**	**Very negative**	**n**^**a**^
Communication/contact with herders	0	1	3	4	1	9
Visits/control of corrals/slaughter	0	0	2	3	4	9
Workload	0	0	7	1	1	9
Total	0	1	12	8	6	

^a^Response rate

When asked how the pandemic affected their lives (Q12, S1.1), two herders stated that too few people were working in the corrals. Four herders pointed to closed national borders (between Sweden and Norway) as problematic for different reasons: access to cheaper groceries, visiting family, and working across the border. Moreover, two herders stated that the negative consequences were related to reduced access to meat markets. Another stated that.

‘[It] has not been time for anything else because of the food crisis, the winter leads to a lot of extra work in the corral.” (Herder #11)

Moreover, one herder claimed that a consequence of the pandemic has been a loss of cultural learning.

### Information Concerning COVID-19

Ten herders (62.5%) were dissatisfied with the governmental distribution of information concerning COVID-19 and control measures for the reindeer husbandry (Q10, S1.1). In contrast, only one (12.5%) of the employees in the management system was dissatisfied with the governmental distribution of information to the management system (one did not want to answer this question, Q8, S1.2). Among the herders that were not satisfied with the information, three reported that there was no or little information. At the same time, two more pointed out that information was hard to find or that they had to request the County Governor office for information. Two herders expressed concerns about missing information concerning what to do if someone got infected:

‘No measures in case of a potential infection. What do we do?” (Herder #16).

Moreover, none of the herders had applied for financial aid because of the pandemic. Two herders said they did not need financial aid, while four herders claimed that the reindeer herders' financial aid requirements were unclear.

“There isn’t any sufficient measurable conditions or measures for the reindeer husbandry.”

(Herder #1)’.

While most employees in the management system were satisfied with the information they received from the central Government, two made comments on what could have been done differently. One participant stated that they lacked information from the central Government concerning how the reindeer husbandry was affected by the regulations, while another stated that “[The central Government] might have provided information at an earlier time.” (Employee #5).

### Infection Control Measures

Nine herders reported that they had taken measures related to infection control in their *siida* or district (Q15, S1.1), while five reported that no measures had been implemented. Of the measures that were implemented, four herders reported that fewer people worked together or that they kept a greater distance between each other, while two reported that counting reindeer or board meetings were cancelled or postponed. Other measures include reducing contact with individuals outside of the reindeer husbandry, not using hired helpers, and washing hands more frequently. Among the herders that had not taken any measures, one stated that there are “few people in the district, feels like family” (Herder #9), while another stated that they had not received information on how to take measures. Additionally, seven herders have changed how they cooperate or communicate with other members in their *siida* because of COVID-19 or infection control measures (Q16, S1.1). As with the above, these changes concern keeping a distance from other herders, limiting visits, being extra careful, and limiting the number of people present.

When employees in the management system were asked what they had done to ease the challenges related to COVID-19 for the reindeer husbandry (Q11, S1.2), two reported that they had provided information to herders. Three reported that they had less physical contact with herders and increased the use of digital communication (e.g., video meetings). One employee commented that there had been “No assemblies at the corral [initiated] by us, [we have] called off actions like counting [reindeer]” (Employee #1). Moreover, two people reported that they worked from home.

## Discussion

For reindeer herders, the main finding from our pilot study was that COVID-19 had little to no impact on daily work. However, impacts varied by area with work in corral, income, and slaughter being negatively affected while using a substitute herder, transporting reindeer, supplementary feeding, and the health of reindeer were not affected. For employees in the management system, communication/contact with herders and visits/control of corrals/slaughter have been negatively affected, while their workload has not been affected. Another important finding was that employees in the management system and reindeer herders had different views on how relevant information concerning the coronavirus and prevention measures has been communicated by the central Government. Reindeer herders stressed that they lacked information on how to implement preventive measures and how to act in case of an outbreak among the herders, while employees in the management system were generally satisfied with the information provided.

### Consequences of the COVID-19 Outbreak

The coronavirus has had a negative effect on the income of the herders. Thus, our findings fit well with previous research on the effect of COVID-19 on pastoral communities (Griffith et al., 2020; Mohamed et al., 2020; Mtimet et al., 2021). While the reindeer pastoral economy was traditionally based on reindeer products (Vorren, 1978), it has shifted towards an increased meat and market adaptation and sedentarization (Riseth, 2006). When pastoralists’ economy becomes more market oriented, they will likely be more affected by national and global economic trends than a less market integrated economy, thus, making herders more vulnerable in a pandemic context compared to more subsistence-based pastoralists. However, it is crucial to notice that Hausner et al. (2011) found that 60% of reindeer herders reported that wages from spouses working outside the reindeer husbandry represent an essential part of household income. In effect, herders who rely on income from outside the reindeer husbandry might be more economically resilient when a crisis like the COVID-19 pandemic occurs.

Surprisingly, most herders report that the pandemic did not affect herd migration. This contrasts with other studies that suggest that the loss of mobility has been the pandemic's most devastating consequence. The fact that reindeer herders reported little change in work related to mobility (e.g., migration from winter to summer pastures) might partly be explained by the unique position that the reindeer husbandry was given when the Norwegian government implemented restrictions. That is, reindeer husbandry is considered a primary food producer and thus as having a critical societal function. As a result, reindeer husbandry was exempted from some of the restrictions introduced in Norway, including access to herding cabins and permission to cross the border to Sweden and Finland – if the movement of the herd demands it – without imposing quarantine (Ministry of Agriculture & Food, 2020b). Pastoralists elsewhere have, in contrast, reported a negative effect of curfews and closed borders, cutting them off from pastures (Food and Agricultural Organization of the United Nations, 2020). However, Mohamed et al. (2020) found that pastoralists who could find a way to continue herding the animals and producing livestock products had suffered less than others who lost jobs and have been confined to their homes. Thus, the exemption reindeer herders have had from some national restrictions might have put them in a better position than pastoralists in other parts of the world.

Less surprisingly, herders reported that the pandemic had a negative effect on working in corrals and for slaughtering reindeer. Working in corrals (including slaughtering) is often a collective endeavour: during calf marking – starting in late June – the reindeer are gathered in corrals, with individual marks cut into the animals’ ears by the respective owner. Similarly, before winter or summer migration, herds are often gathered in corrals to separate mixed herds. Thus, corral work often includes many individuals from several families in the same *siida* and/or from different *siidas*, which was discouraged in the national guidelines.

Surprisingly, the management system employees ranked the coronavirus's general effects as slightly more negative for the reindeer husbandry than the herders reported. One explanation for this difference might be related to the different points of view of the two roles. Individuals working within the management system might have a broader impression of the state of the reindeer husbandry across and between the different counties. Consequently, employees in the management system might have a better overview of the coronavirus's overall effect and the preventive measures, while the herders' view might be more restricted to the situation in their *siida* or district.

### Status of Information and Preventive Measures

Most herders reported a lack of information concerning preventive measures related to the coronavirus. Moreover, there was a different perception between the employees in the management system and reindeer herders concerning the dissemination of relevant information from the central government. That is, while most herders reported that they had received insufficient information, most of the management system employees were satisfied with the information. This is not surprising since employees in the management system have more direct access to information disseminated by the central government than herders. However, in Troms and Finnmark county, the first instructions targeting reindeer herders were published on the 21st of December 2020, more than nine months after the first national lockdown. In contrast, the County Governor in Troms and Finnmark published information to farmers on the 3rd of April 2020 (County Governor of Troms & Finnmark, 2020b).

Most herders reported that they had taken measures related to the coronavirus. However, among the herders who had not taken any measures, lack of relevant information was the main reason. This lack of information concerning procedures might have severe consequences in the event of an outbreak, both concerning personal life, economy, and animal welfare if the herders are unable to carry out their responsibilities. Significantly, historic and contemporary discrimination among ethnic minorities has led to distrust in social institutions, which might harm the trust in – and willingness to adapt to – safety measures concerning public health information (Bavel et al., 2020:463). In effect, Bavel et al., (2020:463) suggest that these communities need more targeted information from trusted organizations. This reflects a significant problem with the ongoing pandemic, the emergence of conspiracy theories concerning the virus. Among the Nenets, reindeer pastoralists in Russia, an accumulation of disasters combined with fewer connections and guidance from their spirits have made them more dependent on inadequate information from the state (Stammler & Ivanova, 2020). Thus, the lack of spirits to guide them through disasters might have led the Nenets to believe in conspiracies that icing of pastures, anthrax outbreaks, and COVID-19 were fabricated to reduce the population on the tundra (Stammler & Ivanova, 2020). This demonstrates the importance of having information that is available, reliable, and quickly disseminated. However, the apparent lag between COVID-19 related instructions for farmers and reindeer herders indicates that this has not been the case during this crisis.

## Concluding Remarks and Implications for Future Studies

While the direct health impact of the virus on the Norwegian reindeer husbandry appears to be limited, the effect on the economy and daily operations seems clear. The corona pandemic has had an enormous impact on societies worldwide and is unlikely to be the last of its kind. Increased air and ocean temperatures over the last century have had a considerable effect on the epidemiology of infectious diseases (Bett et al., 2017). Moreover, pastoralists and their livestock live in constant interaction with each other, with the livestock often in direct contact with wildlife, thus presenting a high threat for exposure to new emerging infectious diseases (Hassell et al., 2020). An upheaval such as the coronavirus outbreak shows the importance of a management system that can provide sufficient and reliable information for areas affected by the crisis.

Our pilot study revealed that the relationship between reindeer herders and employees in the management system needs more attention in the future. This despite several shortcomings, notably relatively small response rate and a relatively large difference in the response rate between reindeer herders (3%) and employees in the management system (16%). One of the reindeer herders was critical to the survey's design: “In the same manes as the preventive measures, this survey is not well-suited for the reindeer husbandry” (Herder #1). Thus, further studies might benefit from in-depth interviews, which was not an option considering the ongoing pandemic. Furthermore, COVID-19 coincided with a forage crisis that arose from extreme snow during the winter and spring of 2020. This might have had several impacts not covered in this pilot study. First, the forage crisis might have been more palpable, thus taking up more resources from the employees in the management system than the pandemic. As a result, the government granted 20 million NOK in financial aid due to the forage crisis (County Governor of Troms & Finnmark, 2020a). Second, the forage crisis might seem more threatening/acute than the threat of COVID-19 and might thus have influenced both herders’ and employees’ responses.

Nevertheless, as the coronavirus continues to spread worldwide, there is an urgent need for knowledge that might mitigate the consequences of the pandemic, both among the Norwegian reindeer herders and pastoralists across the globe.

## Supplementary Information

Below is the link to the electronic supplementary material.

Supplementary file1 (DOCX 36 KB)

## Footnotes

1 Numbers extracted from WHO (https://www.who.int/publications/m/item/weekly-epidemiological-update-on-covid-19---13-july-2021) 16 July 2021.

2 In unpublished data from interviews with tamreinlag reindeer herders, it was claimed that when they first started reindeer husbandry, Saami herders were hired to teach them how to herd reindeers. The same information can be found on villrein.no (https://www.villrein.no/aktuelt/nordfjella-eldste-omrdet-for-tamreindrift-i-sr).

3 The Norwegian Agriculture Agency does not provide statistics concerning the number of people involved in this form of reindeer herding.

4 The County Governor in Trøndelag also has jurisdiction on the reindeer husbandry connected to the south of Trøndelag, i.e., the Saami reindeer husbandry in Innlandet county and the tamreinlag with pastures in both Innlandet and Vestlandet and Viken counties.

5 While the County Governor in Troms and Finnmark and Trøndelag provide the number of employees in the section for rendeer herding, Nordland only provides the number for the section of Agriculture and Reindeer Husbandry (30 persons).

6 NSD seems to be stricter concerning the use of Web survey platforms than the European Union’s General Data Protection Regulation (GDPR) (https://ec.europa.eu/info/law/law-topic/data-protection_en). While the use of e.g., Google does comply with GDPR it does not comply with NSD.

7 Within the timeframe of this pilot, it was impossible to design an online study that both addressed the overall research question and NSDs requirements. For example, we were asked to not ask questions concerning having contracted COVID-19, knowing someone having contracted COVID-19, being at risk or living with someone at risk if contracting COVID-19 as this was considered ‘health personal sensitive’ related information that could be linked by IP addresses, and, thus from NSD’s point of view not necessary to ask about. More generally, Hansen and Fürst (2021), argue that it is paradoxical when specific individuals and groups are categorized – based on definitions of vulnerability or sensitivity – as so research ethically problematic to research that they are excluded from relevant research projects. For example, while NSD originally was meant to only provide advice and guidelines, almost all anthropologists in Norway experience that NSD “… give green and red lights for what is acceptable research, that they actually have decision-making authority” (Hansen & Fürst, 2021:98, translated from Norwegian). To justify the overall aim of investigating the effect of COVID-19 pandemic we thus had no choice but to send the survey by mail.

8 For example, to ensure that the surveys used in this project were completely anonymous, NSD required that no geographical information could be collected, nor any information that could separate the Saami reindeer husbandry from the tamreinlag.
