# Supplementary material for: Consequences of COVID-19 on the Reindeer Husbandry in Norway: a Pilot Study Among Management Staff and Herders
Source: Hum Ecol Interdiscip J. 2022 May 2;50(3):577–88. doi: 10.1007/s10745-021-00295-0 (PMC9059113; doi:10.1007/s10745-021-00295-0)
Supplement: Supplementary file 1 — Supplementary file1 (DOCX 36 KB) [file 10745_2021_295_MOESM1_ESM.docx]

# S1 Supplementary Material: Survey

## S1.1 Reindeer herders

### About you

**1. Gender**

Man

Woman

Other

Don't want to answer

**2. Age:** ________

**3. What role do you have reindeer herding (tick the option that suits you best)?**

Have own siida share

Have own mark, but not siida share

Works as a substitute herder

Other (specify below)

_________________________________________

**4. How long have you had this role (years)?**: ___

**5. Do you have any commission of trust in connection with reindeer herding?**

Yes

Sits on the District/Siida Board

Sits on the reindeer herding board

NRL member

Other (specify below)

_______________________________

No

### General about Covid-19

**6. Do you experience being at risk for Covid-19?**

Yes

No

Don't want to answer

**7. Do you live with someone at risk for Covid-19?**

Yes

No

Don't want to answer

**8. Have you been diagnosed with Covid-19?**

Yes

No

Don't want to answer

**9. Do you know anyone in the reindeer husbandry who has been diagnosed with Covid-19?**

Yes

No

Don't want to answer

If **yes,** what relationship do you have with the person?

Close family

Friend

Works in the same siida

**10. Do you think that information about infection control measures and Covid-19 has been satisfactorily communicated from the authorities to the reindeer husbandry?**

Yes

No

Elaborate on no: __________________________________

Don't want to answer

**11. How do you think covid-19 and infection control measures have affected reindeer herding?**

***Either*:**

No consequenses

| ***Or*:** | 1 | 2 | 3 | 4 | 5 |  |
| --- | --- | --- | --- | --- | --- | --- |
| Positively | ○ | ○ | ○ | ○ | ○ | Negatively |

**12. How have Covid-19 and infection control measures affected your life?**

It hasn't had any consequences for me.

It has had very negative consequences

It has had some negative consequences

It has had some positive consequences

It has had very positive consequences

Elaborate consequences: _____________________________

### Covid-19 and the daily work of reindeer herding

The next questions are about how the daily situation in the reindeer husbandry has changed since March 2020.

**13. To what extent has infection and measures affected the following parts of the reindeer husbandry (Check one option per question):**

|  | **No change** | **Very positive** | **Some positive** | **Some negative** | **Very negative** |
| --- | --- | --- | --- | --- | --- |
| Use of substitute herder | ○ | ○ | ○ | ○ | ○ |
| Transportation of animals | ○ | ○ | ○ | ○ | ○ |
| Work in corral | ○ | ○ | ○ | ○ | ○ |
| Supplemenatry feeding | ○ | ○ | ○ | ○ | ○ |
| Migration | ○ | ○ | ○ | ○ | ○ |
| Slaughter | ○ | ○ | ○ | ○ | ○ |
| Reindeer health | ○ | ○ | ○ | ○ | ○ |
| Reindeer herding tourism | ○ | ○ | ○ | ○ | ○ |
| Income from reindeer husbandry | ○ | ○ | ○ | ○ | ○ |
| Other: _______ | ○ | ○ | ○ | ○ | ○ |

**14. Have you received any financial support in connection with Covid-19?**

Yes

Explain: ____________________________

No

Explain: ____________________________

**15. Has your siida/district initiated infection control measures in connection with day-to-day operations?**

Yes

Explain: __________________________________

No

Explain: __________________________________

**16. Has the way you cooperate or communicate with other members of your siida changed with regard to corona or infection control measures?**

Yes

Explain: __________________________________

No

Explain: __________________________________

That was the end of the survey. Thank you so much for participating!

If you have any comments on the survey, you can write them down here:

## S1.2 Employees in the management system

### About you

**1. Gender**

Man

Woman

Other

Don't want to answer

**2. Age:** ________

**3. What position do you have in reindeer herding management?** ______________

### General information about Covid-19

**4. Do you experience being at risk for Covid-19?**

Yes

No

Don't want to answer

**5. Do you live with someone at risk for Covid-19?**

Yes

No

Don't want to answer

**6. Have you been diagnosed with Covid-19?**

Yes

No

Don't want to answer

**7. Do you know anyone in reindeer herding or management system who has been diagnosed with Covid-19?**

Yes

No

Don't want to answer

If yes, what relationship do you have with the person? ______________________

**8. Do you think that information about infection control measures and Covid-19 has been satisfactorily communicated to the reindeer herding management?**

Yes

No

Elaborate on no: __________________________________

Don't want to answer

**9. How do you think covid-19 and infection control measures have affected *the reindeer husbandry?***

**Either:**

No influence

| **Or:** | 1 | 2 | 3 | 4 | 5 |  |
| --- | --- | --- | --- | --- | --- | --- |
| Positively | ○ | ○ | ○ | ○ | ○ | Negatively |

**10. How do you think covid-19 and infection control measures have affected** the ***management*** **of reindeer herding?**

**Either:**

No influence

**Or:**

|  | 1 | 2 | 3 | 4 | 5 |  |
| --- | --- | --- | --- | --- | --- | --- |
| Positively | ○ | ○ | ○ | ○ | ○ | Negatively |

**11. What measures have you taken** **to relieve challenges related to Covid-19 for reindeer herders?**

**_______________________**

**12. To what extent has infection and measures affected your everyday work (tick an option per question)?**

|  | **No change** | **Very positive** | **Some positive** | **Some negative** | **Very negative** |
| --- | --- | --- | --- | --- | --- |
| Communication/contact with reindeer herders | ○ | ○ | ○ | ○ | ○ |
| Visit/control of facilities/corral/slaughter | ○ | ○ | ○ | ○ | ○ |
| Workload? | ○ | ○ | ○ | ○ | ○ |
|  |  |  |  |  |  |
| Other: _______ | ○ | ○ | ○ | ○ | ○ |

If you have any comments on the survey, you can write them down here:
